# Glycoconjugate vaccines against *Salmonella enterica* serovars and *Shigella* species: existing and emerging methods for their analysis

**DOI:** 10.1007/s12551-021-00791-z

**Published:** 2021-04-10

**Authors:** Aleksandra Bazhenova, Fang Gao, Barbara Bolgiano, Stephen E. Harding

**Affiliations:** 1 School of Biosciences, University of Nottingham, Sutton Bonington, Loughborough, LE12 5RD UK; 2 Division of Bacteriology, National Institute for Biological Standards and Control (NIBSC), Blanche Lane, South Mimms, Potters Bar, EN6 3QG UK; 3 Museum of Cultural History, University of Oslo, Postboks 6762 St. Olavs plass, 0130 Oslo, Norway

**Keywords:** Capsular polysaccharide, Enteric fever, HPAEC-PAD, O-Antigen, Typhoid, Vi

## Abstract

The global spread of enteric disease, the increasingly limited options for antimicrobial treatment and the need for effective eradication programs have resulted in an increased demand for glycoconjugate enteric vaccines, made with carbohydrate-based membrane components of the pathogen, and their precise characterisation. A set of physico-chemical and immunological tests are employed for complete vaccine characterisation and to ensure their consistency, potency, safety and stability, following the relevant World Health Organization and Pharmacopoeia guidelines. Variable requirements for analytical methods are linked to conjugate structure, carrier protein nature and size and *O-*acetyl content of polysaccharide. We investigated a key stability-indicating method which measures the percent free saccharide of *Salmonella enterica* subspecies *enterica* serovar Typhi capsular polysaccharide, by detergent precipitation, depolymerisation and HPAEC-PAD quantitation. Together with modern computational approaches, a more precise design of glycoconjugates is possible, allowing for improvements in solubility, structural conformation and stability, and immunogenicity of antigens, which may be applicable to a broad spectrum of vaccines. More validation experiments are required to establish the most effective and suitable methods for glycoconjugate analysis to bring uniformity to the existing protocols, although the need for product-specific approaches will apply, especially for the more complex vaccines. An overview of current and emerging analytical approaches for the characterisation of vaccines against *Salmonella* Typhi and *Shigella* species is described in this paper. This study should aid the development and licensing of new glycoconjugate vaccines aimed at the prevention of enteric diseases.

## Introduction

According to a systematic analysis performed for *The Global Burden of Disease Study* (GBD) in 2019, the diarrhoeal disease was ranked as the third leading cause of mortality in children and the ninth among all age groups worldwide, contributing to a total of 1.53 million deaths per year (Global Burden of Diseases 2019; Global Burden of Diseases 2019 Risk Factor Collaborators 2020). Bacterial pathogens such as *Salmonella* subspecies *enterica* serovar Typhi (*S*. Typhi), *Shigella* spp., *E. coli* and rotavirus account for the largest proportion of deaths from all infectious diseases. The fast emergence of antibiotic resistance observed in bacterial enteric pathogens is of global significance (Humphries and Schuetz 2015; Tribble 2017; Khalil et al. 2018). *The Global Antimicrobial Resistance Surveillance System* (GLASS) listed these as priority pathogens in 2018 (WHO 2018).

Estimates from *The GBD Study* suggested that the burden of enteric fevers was around 9.24 million due to *S.* Typhi in 2019 and 14.3 million from *S*. Typhi and *S*. Paratyphi together in 2017. Of 136,000 deaths, the vast majority of deaths (86%) were due to typhoid fever from *S.* Typhi, of which 67% of deaths occurred in South Asia (Stanaway et al. 2019). Young children and endemic populations for these pathogens in South and Southeast Asia and sub-Saharan Africa are at the most risk. *S.* Paratyphi A results in the most enteric fever cases in Asia, while bacteraemia in children in Africa is mainly caused by *S.* Typhimurium and *S.* Enteritidis (MacLennan et al. 2014). The main risk factors are unsafe water, inadequate hand hygiene and poor sanitation (Global Burden of Diseases 2019; Global Burden of Diseases 2019 Risk Factor Collaborators 2020). High prevalence of the disease in low- and middle-income countries (LMICs), as well as its spread into the wider population highlight the urgent need for effective protection.

In addition to *S.* Typhi and *S.* Paratyphi, other *Salmonella enterica* serovars are responsible for a high burden of disease-causing death and morbidity globally (0.6 to 3.4 million cases annually, especially among children in sub-Saharan Africa (MacLennan and Steele 2019). The most important etiological agents of invasive nontyphoidal *Salmonella* (iNTS) disease are *S.* Typhimurium and *S.* Enteritidis. Nontyphoidal *Salmonella* are responsible for up to 29% of community-acquired (non-malarial) bloodstream infections in sub-Saharan Africa with an average case fatality rate of 15–20% (Reddy et al. 2010; MacLennan and Steele 2019). Also, for these pathogens, the effectiveness of antibiotic treatment is hampered by the difficulty in making a precise diagnosis, the sudden and rapid onset of the disease, and the growing levels of multidrug resistance.

*Shigella* is the second leading cause of diarrhoeal disease-related mortality after rotavirus and a primary cause of mortality in individuals older than 5 years old (Troeger et al. 2018). With an annual burden of 165 million cases mostly in developing countries, shigellosis accounted for 213,000 deaths in all age groups worldwide, 30% of which were children < 5 years old (Khalil et al. 2018; WHO 2018). Shigellosis often leads to complications like dysentery that needs antibiotic treatment, which is an emerging challenge due to its resistance. The current WHO recommendations to treat shigellosis include fluoroquinolones as a first line and β-lactams and cephalosporins as the second line with almost no alternative medicines available (Williams and Berkley 2016; Cohen and Muhsen 2019).

Since capsular polysaccharides and serotype-specific O-polysaccharides are tightly linked with the virulence of enteric pathogenic bacteria, global efforts are being taken to develop vaccine candidates using these antigens to provide long-term antibody-mediated protection from enteric diseases (Cohen and Muhsen 2019). Low immunogenicity of many bacterial glycans can be compensated via carrier protein conjugation that helps to induce a T-cell-dependent response (Rappuoli et al. 2018; Micoli et al. 2018a, b, c; MacCalman et al. 2019; Berti and Micoli 2020). Poor immunogenicity and heterogeneity of bacterial polysaccharides make glycoconjugate vaccine development a challenging but important task, as early successes show. Vaccines can become a rapid solution for the control of enteric disease in both LMICs and high-income countries. To reach the people in need, these vaccines must be licensed in accordance with WHO recommendations, national or regional pharmacopoeias and specific approvals issued by the National Regulatory Authorities (NRAs). From the early development stage, the identity, purity, physico-chemistry, stability, composition, size, efficacy and safety profiles of glycoconjugate vaccines must be analysed and meet standards set by the NRA (Ravenscroft et al. 2015b). The global prevalence of these enteric pathogens and the unmet need for effective and accessible vaccines reflect the demand for more rapid and precise characterisation methods that would provide superior quality control in line with existing regulations. This paper reviews current and emerging approaches to vaccine design and analytical methods aiding development and quality control of glycoconjugate vaccines based on Vi capsular polysaccharide of *S.* Typhi and lipopolysaccharide O-antigen (O-Ag) polysaccharide of *Shigella* spp.

## Part I

### *S.* Typhi and *Shigella* spp.: strain diversity and pathogenesis

*S.* Typhi and *S*. Paratyphi and *Shigella* spp. are Gram-negative bacteria, which are responsible for typhoid/paratyphoid fevers and shigellosis, respectively (Riddle et al. 2018). At a relatively low dosage, these pathogens also can cause asymptomatic infections, with *Salmonella* being capable to infect starting at about 10^5^–10^8^ organisms and *Shigella* spp. with a load of only 10 bacteria (Puzari et al. 2018; Liu et al. 2016). *Salmonella enterica* serovars Typhi and Paratyphi A (rarely B or C) are the main cause of disease. In contrast, shigellosis can be caused by representatives of the *Shigella* family, which is subdivided into four groups (Cohen et al. 2019). Within each defined group, multiple serotypes exist (Table 1). Up to 90% of the shigellosis cases are caused by the *S. flexneri* and *S. sonnei* serotypes (Page et al. 2016; Kotloff et al. 2018). *S. flexneri* is mostly endemic in LMICs, while *S. sonnei* tends to be present in developed countries, where it overrides other serotypes. The enhanced ability of *S. sonnei* to develop resistance to broad-spectrum antimicrobials makes it an important target pathogen, especially in developed countries, where it has started to become more prevalent. This serotype can accept horizontally transferred DNA and maintain it with better stability than *S. flexneri*, which results in a growing dominance. DNA transfer results in O-antigen switching, and O-antigens are the main immunogens in vaccines (Anderson et al. 2016; Thompson et al. 2015; Das and Mandal 2019).

**Table 1:** Currently known serotypes of *Shigella* spp.

*Shigella* species	Known serotypes	Newly identified serotypes
*Shigella dysenteriae*	1–15	-
*Shigella flexneri*	1a, 1b, 1d, 2a, 2b, 3a, 3b, 4a, 4av, 4b, 5a, 5b, 6, X, Y	Xv, Yv,7a, 7b
*Shigella boydii*	1–19	-
*Shigella sonnei*	1	-

Adapted from Muthuirulandi Sethuvel et al. (2016)

Complications of the infection include intestinal immunosuppression that may overpower the established immunity to *Shigella* giving rise to repeat infections (Brunner et al. 2019). Diversity among pathogenic serotypes, high infectious capacity, variations among clinically relevant strains especially within the *S. flexneri* family, and disease seasonality and geographic distribution and prevalence complicate the development of an effective vaccine (Muthuirulandi Sethuvel et al. 2016; Knirel et al. 2015; Barel and Mulard 2019; Das and Mandal 2019)*.*

### Antibiotic resistance

Recent reports alert on rising extensive drug resistance (XDR) in *S.* Typhi to common antimicrobial agents, such as co-trimoxazole, ampicillin, chloramphenicol and trimethoprim-sulfamethoxazole, fluoroquinolones and third-generation cephalosporins (Klemm et al. 2018). In endemic populations with high prevalence of XDR *Salmonella*, not many alternative treatments exist apart from azithromycin and carbapenems (Klemm et al. 2018). But today, bacteria are still capable of developing resistance to azithromycin when used as a second-line agent (Ahsan and Rahman 2019). Emerging advantageous genetic mutations for pathogen survival and ciprofloxacin resistance (recommended as first-line treatment) were recently reported by Pathogenwatch. With increasingly limited treatment options for *S.* Typhi, control strategies like vaccination and hygiene maintenance must be established (Argimón et al. 2020).

In *Shigella*, multiple studies have found rising resistance to ciprofloxacin, and quinolones, especially among isolates from children (WHO 2005; Williams and Berkley 2018). Moreover, in 2014, WHO published a detailed report on the geographical burden of fluoroquinolone resistance among *Shigella* isolates, which showed worrying rates especially for the South-East Asia region which accounts as one of the endemic regions for shigellosis (Table 2) (WHO 2014a). A retrospective review showed that multidrug resistance among *Shigella* isolates varies from 36–98% (Muthuirulandi Sethuvel et al. 2016). Despite this, some of these antibiotics remain in the official guidelines globally (Table 3). The *Infectious Diseases Society of America* (IDSA) strongly recommended precise diagnostic tests and strict adherence to public health policies when working with patients suffering from enteric infections in high transmission risk areas and advised cautious prescription of antimicrobial agents (Shane et al. 2017). For example, the WHO recommendations for treatment of dysentery had minimal alterations from 2005 to 2013 and feature ciprofloxacin as the first-line antibiotic and pivmecillinam, ceftriaxone and azithromycin as second-line options. Note that pivmecillinam has no paediatric formulation and azithromycin is not recommended for paediatric treatment, while ceftriaxone is recommended for injection in children and not adults (WHO 2005).

**Table 2:** Collated data on *Salmonella* and *Shigella* fluoroquinolones resistance

Reporting region (data based on > 30 tested isolates)	Range of resistant proportion in isolates (%)	
	Nontyphoidal *Salmonella*	*Shigella*
African region		
- National data	0–35	0–3
- Publications	0–30	0–9
Region of Americas		
- National data	0–96	0–8
- Publications	0	0–20
Eastern Mediterranean		
Region		
- National data	2–49	3–10
- Publications	0–46	0–41.3
European Region		
- National data and reports to	2–3	0–47
FWD-Net		
- Publications	13	0
South-East Asia Region		
- National data	0.2–4	
- Publications	1.4	0–82
Western Pacific Region		
- National data	0–14	3–28
- Network/institution data	0–0.3	0
- Publications		2

Adapted from the World Health Organization Antimicrobial Resistance Global Report on Surveillance (2014a). *FWD-Net*, Foodborne and Waterborne Diseases and Zoonoses Network

**Table 3:** Existing Guidelines for treating traveller’s diarrhoea and dysentery

Organisation issuing the guidelines and year issued	Condition or causative pathogen	Age group/specifications	Treatment options	Comments
Infectious Diseases Society of America (Shane et al. 2017)	Infectious diarrheal disease	Adults and children Children	Fluoroquinolones: ciprofloxacin or azithromycin (based on susceptibility patterns and travel history) 3rd generation cephalosporin for infants < 3 months old or azithromycin	Advice against antimicrobial use upon confirmed detection of bacteria and in immunocompetent patients and against Shiga2 toxin-producing pathogens
WHO Expert Committee EML listings (WHO 2017c)	Invasive bacterial diarrhoea/dysentery	All age groups	First line: ciprofloxacin Second line: ceftriaxone, cefixime, azithromycin, sulfamethoxazole + trimethoprim	Treatment of traveller’s diarrhoea with sulfamethoxazole + trimethoprim in all age groups. Azithromycin and fluoroquinolones to be used as last resort options (resistance and potential harm concerns). For confirmed *Shigella* dysentery ceftriaxone (beta-lactam) was proposed as a superior class of antibiotics.
National Institute for Health and Care Excellence (NICE 2020)	Traveller’s diarrhoea	Adult only	Standby antimicrobial: azithromycin 500 mg for 1 to 3 days Prophylaxis/treatment: bismuth subsalicylate, 2 tablets four times daily for 2 days	No indications on specific treatment against a causative organism provided in the guidelines
Guidelines for the control of shigellosis, including epidemics due to *Shigella* dysenteriae type 1 (WHO 2005)	Dysentery (shigellosis)-*Shigella dysenteriae* type 1	Adults Children Adults Children Children only Adults only	1st line: ciprofloxacin 500 mg/ 15 mg/kg 2 times per day for 3 days, by mouth 2nd line: pivmecillinam 100 mg/ 20 mg/kg 4 times per day for 5 days, by mouth 2nd line: ceftriaxone injection 50–100 (50–80 mg according to the Pocket book of hospital care for children (second edition))-Once a day IM for 2 to 5 days (WHO 2013), only when *Shigella* strains are resistant to ciprofloxacin OR azithromycin 6–20 mg/kg, orally, once daily for 1–5 days Alternative 2nd line cefixime.	Ampicillin. chloramphenicol, co-trimoxazole, tetracycline, nitrofurans, Aminoglycosides, first- and second-generation cephalosporins, amoxicillin, nalidixic acid NOT to be used as treatment options due to resistance or cross-protection effects Pivmecillinam (amdinocillin pivoxil) and ceftriaxone are considered effective against multi-resistant *Shigella* strains in all age groups, but they come at a high price. Co-trimoxazole prophylaxis an important intervention for preventing mortality in HIV-infected and -exposed infants and children (WHO 2010)
Pocket book of hospital care for children (second edition) (WHO 2013)	Dysentery	Children	1st line: ciprofloxacin 15 mg/kg twice daily for 3 days 2nd line: ceftriaxone 50–80 mg/kg for 2–5 days	Trials urgently needed to enable azithromycin as an alternative first-line therapy in children. Trials to investigate cefixime as an alternative option are urgently needed (Williams and Berkley 2016).

### Polysaccharides as target antigens

#### *Salmonella* Typhi capsule

Pathogenic bacteria often produce extracellular polysaccharide capsules that serves as virulence factors in promoting attachment and colonisation and in protecting the bacteria from opsonisation, complement binding and opsonophagocytosis, as well as providing hydration and transport functions. The Vi capsular polysaccharide of *S.* Typhi is immunogenic and is represented by non-stoichiometrically *O*-acetylated α-1,4-linked *N*-acetylgalactosaminuronic acid residues and is crucial for host infection, making it a major vaccine target (Liston et al. 2016). The effectiveness of Vi as an immunogen has been an important research topic that led to a promising route to a safer and more protective vaccine design (Robbins and Robbins 1984).

#### Variability of O-antigen targets in Vi-negative *Salmonella* and *Shigella*

Not all enteric *Salmonella* contain a capsular polysaccharide, leaving the bacterial cell surface-exposed O-Ag as a promising vaccine target. In common with other Gram-negative organisms, the cell envelope of *Salmonella* and *Shigella* contains a lipopolysaccharide (LPS) complex. This consists of an outer O-polysaccharide coat, a middle portion (the R core) and an inner hydrophobic lipid A chain. The LPS moiety may function as an endotoxin and O-polysaccharide repeating units confer O-Ag specificity and virulence (Whitfield et al. 2020). For example, rough serotypes of *Salmonella* and *Shigella* spp. with incomplete or no surface O-polysaccharide are usually avirulent or attenuated compared to the smooth serotypes which have a complete O-Ag moiety. *S.* Typhi serovars are positive for LPS O9 and O12 antigens and Vi capsular polysaccharide (Crump et al. 2015). But, *S.* Typhi may have a Vi-negative serotype, so O-Ag protein conjugation approaches may need to be applied (Salman et al. 2017).

Non-typhoidal *Salmonella* are the representatives of non-encapsulated enteric pathogens that contain LPS as an outer membrane component. While lipid A is an exceedingly conserved element among Gram-negative bacteria, which is responsible for toxicity, the nature and structures of the repeating glycans in the O-antigen chain are often serotype specific and important for pathogenicity. The R core has a proximal inner region composed of heptose residues, where phosphate, phosphorylethanolamine or pyrophosphorylethanolamine groups are often contained as substitutions; the outer core is usually built of neutral or amino hexoses (Whitfield et al. 2020). In glycoconjugate vaccines against iNTS, the core and O-Ag portions of the LPS are used to induce a protective response. Recently, flagellin proteins native to iNTS serovars were tested in the role of secondary antigens and shown to be a promising approach for the development of a highly potent vaccine (Baliban et al. 2018; Baliban et al. 2020).

*Shigella* spp. do not possess a capsular component but all contain LPS anchored to the outer membrane via lipid A. Antigenic O-polysaccharide chains attached at the distal end of the LPS core define the serotype affiliation and strain-dependent immune specificity of *Shigella*. The length and structural availability of this O-Ag play a key role in pathogenesis and host resistance (Lindberg et al. 1991; Caboni et al. 2015). The O-Ag is unique for each serotype, reflecting the genetic diversity of *Shigella* spp. (Perepelov et al. 2012).

## Part II

### Vaccines on the market and in the pipeline

In the 1960s, the first live-attenuated *S.* Typhi vaccine was created and gave up to 7 years of protection (Ashcroft et al. 1967). Nevertheless, systemic adverse events following the use of such vaccines excluded them from modern treatment approaches (Ashcroft et al. 1964). Today, efforts are taken in the direction of native and synthetic polysaccharide formulations, protein-conjugated antigens, combination vaccines and alternative delivery systems like GMMA (generalised modules for membrane antigens) (Rossi et al. 2014). Four Vi conjugate vaccines have been licensed with one so far receiving WHO prequalification. Vi-DT (diphtheria toxoid) and others are in late-stage development or waiting for approval. There are several Vi PS based and a number of bivalent conjugate vaccines against Typhi and Paratyphi in development. Examples can be seen in Table 4.

**Table 4:** Typhoid and paratyphoid vaccines landscape: licensed and in clinical trials

Disease	Vaccine name/construct	Manufacturer	Status
Typhoid	Typbar-TCV/glycoconjugate Vi-TT	Bharat Biotech India Ltd, Hyderabad	Licensed in India, Nepal, Nigeria Licensed, and prequalified by WHO in Dec 2017 for UNICEF procurement (Jin et al. 2017)
Typhoid	PedaTyph/glycoconjugate Vi-TT	Bio-Med Pvt. Ltd, India	Licensure in India (Syed et al. 2020)
Typhoid	ZyVAC-TCV/glycoconjugate Vi-TT	Cadila Healthcare Limited, India	Licensed in India Launched for Typhoid (in adult volunteers) in India Adisinsight.springer.com 2020a, b) WHO prequalification will be sought
Typhoid	Glycoconjugate Vi-TT	Walvax	Licensed in India in 2020 (Steele et al. 2020) Preclinical (Khan et al. 2017)
Typhoid	TYPHIBEV/glycoconjugate Vi-CRM_197_	Biological E Ltd, India/GVGH (GSK) Technology Transfer Agreement (TTA)	WHO prequalification obtained in Dec 2020
Typhoid	Glycoconjugate Vi-DT	SK Bioscience The International Vaccine Institute (IVI TTA)	Phase II completed, enrolling into phase III (Steele et al. 2020)
		PT Bio Farma (IVI TTA)	Phase-III clinical trials in typhoid (in adolescents, in children, in infants, prevention, in adults) in Indonesia (IM) ClinicalTrials.gov Identifier: NCT04051268
		Incepta (IVI TTA)	Preclinical (Adisinsight.springer.com 2020a, b)
Typhoid	Glycoconjugate Vi-TT	Eubiologics, Korea	Phase 1 (Syed et al. 2020)
Typhoid	Glycoconjugate Vi-DT	DAVAC/Finlay Institute, Vietnam	Preclinical (Syed et al. 2020)
Typhoid	Glycoconjugate Vi-PspA	IVI	Preclinical (Kothari et al. 2014)
Typhoid	Typhim Vi/Vi PS	Sanofi Pasteur SA	Prequalified by the WHO (WHO prequalified vaccines list)
Typhoid	Typherix/Vi PS	GSK	Discontinued due to better alternatives available and manufacturing struggles in 2018 (GSK 2018)
Typhoid	Ty21a (Vivotif)/live attenuated	PaxVax	Prequalified by the WHO (Sahastrabuddhe and Saluja 2019)
Paratyphi A and Typhoid	Glycoconjugate O:2,12-TT + Vi-TT	NIH, Lanzhou	Phase II (Martin et al. 2016)
Paratyphi A and Typhoid	Glycoconjugate O:2,12-CRM_197_ + Vi-CRM_197_	GVGH, Biological E	Preclinical (Martin et al. 2016)
Paratyphi A	Glycoconjugate CVD 1902 + CVD 909/ mutations in *guaBA* and *clpX*	University of Maryland Baltimore (UMB), Bharat Biotech	Phase I (Martin et al. 2016)
Paratyphi A	Glycoconjugate O:2,12-DT + Vi-DT	IVI	Preclinical (Martin et al. 2016)
iNTS	Trivalent glucoconjugate (*S. typhimurium* COPS:FliC+ TypbarTCV)	UMSOM-CVD, Bharat Biotech (Hyderabad, India	Preclinical, planned to roll into phase 1/2 (Baliban et al. 2020)
iNTS	*S. typhimurium* LH1160 (ΔpurB + ΔphoP/Q) live-attenuated vaccine	Massachusetts General Hospital, Boston	Phase 1 (Angelakopoulos and Hohmann 2000). Not followed up due to weak response
iNTS	*S. typhimurium* WT05 (Δ*aroC* + Δ*ssaV*) live-attenuated vaccine	Microscience	Phase 1 (Hindle et al. 2002)
iNTS	GMMA (*S. typhimurium* and *S. enteritidis*)	GSK Vaccines Institute for Global Health S.r.l, Siena, Italy	Preclinical (Baliban et al. 2020)
iNTS	*S. enteritidis* and *S. typhimurium* COPS:FliC glycoconjugates	CVD at the University of Maryland School of Medicine, Baltimore, Maryland USA	Preclinical (Baliban et al. 2018)
iNTS	GMMA	GVGH	Preclinical (Martin et al. 2016)

*Salmonella* Typhimurium and *S*. Enteritidis bivalent vaccine could represent a valuable public health intervention. Two approaches, both O-Ag based, have been evaluated. The first approach is based on glycoconjugation, where *S.* Typhimurium and *S*. Enteritidis O-Ag are independently linked to CRM_197_ or a homologous flagellin protein as carriers. The second approach is represented by the development of a bivalent formulation of *S*. Typhimurium and *S*. Enteritidis genetically modified outer membrane vesicles (OMV) termed “GMMA” (Micoli et al. 2018a, b, c). Several glycoconjugates are now in preclinical development for iNTS, and some employ flagellin from *S*. Enteritidis to function as both a carrier protein for bacterial O-Ag and a secondary antigen in an attempt to increase the efficacy. This preparation is scalable and allows bivalent/multivalent formulations for different O-PS antigens. This conjugation strategy may contribute to an enhanced immunogenicity and safety of this potential vaccine candidate and is likely to come at a lower production cost, which is important for implementation in LMICs (MacLennan and Steele 2019; Baliban et al. 2020).

With over 50 serotypes of *Shigella* arising from O-Ag variations, the development of an appropriate target for a vaccine is challenging. Antigen variability complicates the protection of the population in non-endemic areas. Thus, a vaccine should preferably be multivalent covering the most pathogenic *Shigella *species such as *S. flexneri* 1b, 2a, 3a and 6, and *S. sonnei* according to the WHO, and a recent meta-study on antibiotic resistance (Mani et al. 2016; Das and Mandal 2019; Micoli et al. 2018a, b, c; Raso et al. 2020; WHO 2018, 2020a). The global enteric multi-centre study suggests that such a multivalent formulation would cover 72% of *Shigella* strains protecting directly and cross-protect for up to 89% of all strains (Livio et al. 2014). *Shigella dysenteriae* type 1 used to be linked to rapid spread and high mortality rates; however, no epidemic cases were reported in the last 20 years and it is rarely included in serotype-specific vaccines (Kotloff et al. 2018). Several vaccines have now been developed with a few entering phase III clinical trials: most candidates are based on *S. flexneri* 2a LPS conjugates (Table 5). Although *Shigella* vaccines lack WHO guidelines for vaccine development, a recent draft version suggests that quality and safety must be monitored at least to the standard of currently licensed glycoconjugate vaccines (WHO 2020a, b). New standards for *Shigella* are also going to be established soon to aid the development of a vaccine evaluation protocol (NIBSC 2020).

**Table 5:** Vaccines against *Shigella* spp.: current landscape

Disease	Vaccine name/construct	Developer	Status
*Shigella dysenteriae*	Bioconjugate vaccine Sd1-EPA (GVXN SD133)	LimmaTech Biologics AG (Former GlycoVaxyn AG) Schlieren, Switzerland	Phase 1 ClinicalTrials.gov Identifier: NCT01069471
*S. flexneri* 2a	FLEXVAC/Tri-acylated lipid A with LPS derived from “smooth” (Ac_3_-S-LPS) derived from *S. flexneri* 2a (Lyodov and Aparin 2014)	Gritvac, Moscow, Russia	Phase III ongoing Clinical study no. 161 (Rosminzdrav 2020)
*S. flexneri* 2a	Live-attenuated ΔguaBA + Δset , Δsen (CVD1208s) (Toapanta et al. 2018)	CVD at the University of Maryland School of Medicine, Baltimore, Maryland USA, PATH	Phase 2 ClinicalTrials.gov Identifier: NCT01531530
*S. flexneri* 2a	Flexyn2a/recombinant O-PS glycoconjugate *S. flexneri* 2a-EPA bioconjugate vaccine	LimmaTech Biologics AG Schlieren, Switzerland (Kämpf et al. 2015)	Phase 2b ClinicalTrials.gov Identifier: NCT02646371
*S. flexneri* 2a	Artificially combined Invaplex_AR_/intranasal macromolecular complex (LPS + IpaC + IpaD proteins)	PATH and WRAIR, Silver Spring, Maryland	Phase 1/2b ClinicalTrials.gov Identifier: NCT02445963 Plans to collaborate with Enesi Pharma to deliver vaccine needle-free
*S. flexneri* 2a	Invaplex_DETOX_ IM (Detoxified LPS + IpaC + IpaD proteins)	PATH, DFID and WRAIR, Silver Spring, Maryland	Phase 1 completed ClinicalTrials.gov Identifier: NCT03869333
*S. flexneri* 2a	*S. flexneri* 2a-TT15 synthetic O-PS based conjugate	Institut Pasteur, Paris, France	Phase 1 ClinicalTrials.gov Identifier: NCT02797236
*S. flexneri* 2a	DB fusion subunit candidate/Ipa proteins (IpaB and IpaD) of Shigella (Martinez-Becerra et al. 2013)	PATH, Washington, DC	Preclinical
*S. flexneri* 2a	Trivalent killed whole-cell Shigella vaccine (Kaminski et al. 2014)	WRAIR, PATH	Preclinical
*S. flexneri* 2a	OMV with heat-inactivated (HT-ΔtolR) mutation (Pastor et al. 2018)	University of Navarra, Navarra, Spain	Preclinical
*S. flexneri* 2a and *S. sonnei*	Oral live F 2a-sonnei (FS) vaccine	China	Licensed (Wang 2003)
*S. flexneri* 2a*+ S. sonnei*	O-PS-rEPA chemical conjugates (monovalent formulation for each strain)	NICHHD (National Institute of Child Health and Human Development), USA	Phase 3 ClinicalTrials.gov Identifier: NCT00368316
*S. flexneri* 2a and *S. sonnei* (potential cross-protection)	Killed whole-cell/O-antigen polymerase mutant, truncated Shigella (Kim et al. 2018)	International Vaccine Institute, Seoul, Korea and PATH	Preclinical. Plan to move into Ph1/2b trials
*S. flexneri* 2a and 3a and *S. sonnei*	CombiVax/live, genetically attenuated typhoid Ty21a with biosynthetic Shigella sonnei O-Ag gene insertion (Dharmasena et al. 2016)	Protein Potential LLC, Rockville, Maryland USA	Preclinical
Four most epidem. relevant strains (cross-protection)/no details	Tetravalent *Shigella* bioconjugate containing four different O-Ag	LimmaTech Biologics AG Schlieren, Switzerland/GSK Vaccines Institute for Global Health	Phase 1/2 ClinicalTrials.gov Identifier: NCT04056117
*S. sonnei*	Live-attenuated vaccines: (WRSS1 (ΔvirG); (WRSs2 (ΔvirG + Δset, Δsen); WRSs3 (ΔvirG + Δset, Δsen, ΔmsbB)	Walter Reed Army Institute of Research (WRAIR), Silver Spring, Maryland	Phase 2b (WRSs2/NIAID) ClinicalTrials.gov Identifier: NCT04242264
*S. sonnei*	4-component GMMA-based 1790GAHB vaccine (Rossi et al. 2014)	GSK Vaccines Institute for Global Health S.r.l, Siena, Italy	Phase 2

### Factors that play a role in immunogenicity for Vi and *Shigella* O-Ag glycoconjugates

#### Antigen chemistry and *O*-acetyl content and modifications

The structural implications of glycan antigens to become an efficient immunogen are still unclear. Apart from carbohydrate chain length, other important viability properties affect the resultant antigen success: the stereochemistry of glycosidic linkages, glycan position within a repeating unit, locations of branching points and biochemical composition, which complicate design and analysis. Size/span of the epitope, terminal glycan residues, presence/absence of branching points, substituent groups, such as *O*-acetyl, and number of repeating units may be considered as key parameters to measure (Anish et al. 2014; Berti et al. 2018). Table 6 lists the repeating unit structures of *S.* Typhi capsular polysaccharide and the O-antigens of vaccine-relevant *Salmonella* and *Shigella* species.

**Table 6:** Structures of the repeating units of capsular polysaccharide and serotype-specific O-Antigens of *Salmonella* serovars and *Shigella* species

Polysaccharide or O-Ag	Repeating unit
*Salmonella* serovars:	
*S.* Typhi Vi	→4) -α-D-GalNAcA(3OAc)-(1→
*S.* Paratyphi A	[α-D-Par 1→3] → 2)-α-D-Man (1→4)-α-L-Rha(2/3OAc)(1→3)-[α-D-Glc(1→6)]-α-D-Gal)-(1→
*S.* Typhimurium (O:4,5)	[α-D-Abe(2OAc) 1→3] → 2)-α-D-Man(1→4)-α-L-Rha(2/3OAc)(1→3)-[α-D-Glc1→4/6]-α-D-Gal-(1→
*S.* Enteriditis (O:9)	[α-D-Tyv 1→3] → 2)-β-D-Man-(1→4)-α-D-Rha-(1→3)-[α-D-Glc]n 1→4]-α-D-Gal-(1→
*Shigella* species:	
*S. dysenteriae* 1	→ 3)-α-L-Rha-(1→3)-α-L-Rha-(1→2)-α-D-Gal-(1→3)-α-D-GlcNac-(1→
*S. flexneri* 1b	→ 2)-α-L-Rha(3/4OAc)-(1→2)-α-L-Rha-(1→3)-α-L-Rha(2OAc)-(1→3)-**[**α-D-Glc→4**]**-β-D-GlcNac-(1→
*S. flexneri* 2a	→ 2) -α-L-Rha(3/4OAc)-(1→2)-α-L-Rha(1→3)-[α-D-Glc-→4]-α-L-Rha-(1→3)-β-D-GlcNAc(6OAc)-(1→
*S. flexneri* 3a	[α-D-Glc→3] →2-α-L-Rha-(1→2)-α-L-Rha-(1→3)-α-L-Rha(2OAc)-(1→3)-β-D-GlcNAc(6OAc)-(1→
*S. flexneri 6*	→2)-α-L-Rha(3/4OAc)-(1→2)-α-L-Rha-(1→4)-β-D-GalA(1→3)β-D-GalNac-(1→
*S. sonnei*	→ 4)-α-L-AltNAcA-(1→3)-β-FucNAc-4-N-(1→

Square brackets denote branched residues. Structures adapted from Heyns and Kiessling (1967) (Vi); Ravenscroft et al. (2015a) (Paratyphi); De Benedetto et al. (2017a) (non-typhoidal *Salmonella*); Liu et al. (2008); Perepelov et al. (2012) (*Shigella*)

For Vi PS, *O*-acetylation is a major factor for immune potency (Szu et al. 1991; Hitri et al. 2019). Vi PS is variably *O*-acetylated at the carbon 3 of the repeating monosaccharide. The *O*-acetyl groups are the most solvent exposed (Szu et al. 1991; Hitri et al. 2019) and the primary epitope exposed for antibody binding since carboxyl and *N*-acetyl groups are buried within the PS helix, making them less available as antibody targets, which are only be exposed in de-*O*-acetylated Vi (Hitri et al. 2019). Antibodies reacting with carboxyl and/or *N*-acetyl groups in de-*O*-acetylated Vi have been reported, but these groups are unlikely to be dominant in Vi vaccines, which meet the recommended level of *O*-acetylation (Qadri et al. 1990; Szu et al. 1991). The relatively high viscosity of Vi makes it more challenging to characterise with physicochemical methods like HPLC-SEC or matrix-based sample separation methods, for example ultrafiltration membranes or solid-phase extraction cartridges in a free polysaccharide assay (Hitri et al. 2019). Partially, *O*-acetylated PS may be structurally beneficial in a formulation to give access to additional epitope(s) (Hitri et al. 2019). Nevertheless, the key to an effective Vi vaccine is to ensure that the *O*-acetyl content will be at least 52% or 2.0 mmol/g of PS as specified by WHO recommendations (Szu et al. 1991; Lemercinier et al. 2000; WHO 2014b).

With iNTS and *Shigella*, the level and position of *O*-acetylation of the O-Ag and any chemical modifications of the LPS in the infectious strains mediate the strength of the immune response and are considered to be important variables for vaccine safety and efficacy (Kubler-Kielb et al. 2007; Giardina et al. 2005). The O-polysaccharide portion of the LPS is the most exposed and is extremely diverse in chain length, chain distribution and composition, which all affect virulence (Carter et al. 2007; Morona et al. 2003; West 2005). The O-Ag can be composed of multiple repeating units (RUs), which contain two to six monosaccharides per RU and are heterogeneously distributed on the LPS molecule. The chain numbers and lengths are serotype dependent (Barel and Mulard 2019). Techniques currently used for characterisation of LPS and O-Ag in vaccine preparations are 1D and 2D NMR, GC, GC-MS and HPAEC-PAD (Micoli et al. 2014; Gerke et al. 2015; De Benedetto et al. 2017a; Raso et al. 2020).

#### Carrier proteins and conjugation chemistry

To confer immunogenicity, the saccharide component is chemically or biologically conjugated to a carrier protein. Currently licensed carriers of Vi PS include tetanus toxoid (TT), diphtheria toxoid (DT) and cross reacting material 197 (CRM_197_), while other glycoconjugates utilise *Haemophilus* protein D and outer membrane protein complex from serogroup B meningococcus and pneumococcal proteins. The recombinant non-toxic form of *Pseudomonas aeruginosa* exotoxin A (*r*EPA) has been evaluated as a carrier protein with *Shigella* antigens and Vi PS (Cohen et al. 1997; Kubler-Kielb et al. 2007; Thiem et al. 2011; Kämpf et al. 2015). Most emerging protein carriers are recombinant, and alternatives such as OMV, GMMA, inorganic nanoparticles and virus-like particles are being investigated. The GMMA approach may be promising for vaccine development in LMICs if it is cheaper to produce and its efficacy meets accepted standards. More methods like one-pot (single reaction mixture) and chemo-enzymatic protocols, along with HPLC-based automated oligosaccharide construction methods and advances in the assembly of synthetic glycan and alternative nanocarriers that are being developed will speed up vaccine development (Safari et al. 2012; Micoli et al. 2018a, b, c; Wen et al. 2018). Such new protocols will also require characterisation, consistency of production and verification of their quality and safety by currently recommended assays.

Glycoconjugate vaccines are produced by either chemical, bioconjugation or GMMA approach, conferring variations in conjugation chemistries that affect the ability to properly define and standardise a molecule. For example, chemical conjugation protocols expose or modify each oligo- or poly-saccharide separately; a similar situation is seen in “sun-type” bioconjugate vaccines with a single point of conjugation. “Lattice-type” conjugates where carrier protein and O-antigens interlink several times may be more challenging to analyse (Barel and Mulard 2019). The integrity of protective saccharide epitopes should be verified following modifications.

Immune responses to vaccines can also be affected by complexity of assembly, linker structure and carrier attachment site number or the structure, conformation and size distribution of the conjugates (Avci et al. 2019). In constructing Vi conjugates, Arcuri et al. discovered that actual conjugation chemistry in terms of linker number conjugated to the carrier protein (in this case CRM_197_) did not impact vaccine immunogenicity as well as did not inter-relate to linker length or the saccharide loading on the carrier protein (2017). While the highest IgG response post-primary dose was achieved with a full-length Vi PS-conjugate versus fragmented, the requirements for an optimal boost varied. For a full-sized antigen, the response was similar irrespective of protein conjugate, but in formulation with fragmented Vi, after second immunisation a booster response was only observed with TT but not with CRM_197_ and DT that may be linked to the larger size of TT as well as its T-cell epitopes (Arcuri et al. 2017; Wessels et al. 1998; Lockyer et al. 2020). With a CRM_197_ conjugate, Vi PS of less than 50 kDa gave a booster dose, while full-length Vi PS led to hypo-responsiveness (Micoli et al. 2020a, b). Although Vi-CRM_197_ and Vi-*r*EPA behaved differently when tested in infants, the source and size of Vi PS were different, and these factors should also be considered (Thiem et al. 2011; Bhutta et al. 2014; Micoli et al. 2020a, b). Another study has also confirmed that immune response to Vi-DT in mice was stronger with an increasing amount of cross-linking and the subsequent size of the conjugate (An et al. 2011). Recently, Sun et al. showed that while conjugation chemistry was not critical for immune response activation, polysaccharide stability and structure dictate the unique antigen presentation method to the T-helper cells, elucidating adaptive immune response (Sun et al. 2018). Moreover, for Vi-CRM_197_, studies in mice indicated that shorter polysaccharide have less risk of inducing unfavourable T-independent immune response and hypo-responsiveness compared to long-chain Vi conjugates. Therefore, the elimination of T-independent responses elucidated by potential glycan candidates may be the next important control used for rational vaccine design and testing (Micoli et al. 2020a, b). For *Shigella*, use of a succinylated carrier protein was found to produce more immunogenic glycoconjugates compared to the unaltered version so chemical modifications of carrier proteins are also important to consider and need to be analysed (Barel and Mulard 2019). These findings highlight that in the development of novel Vi PS vaccines, the PS chain length and the carrier protein of candidate vaccines may need careful monitoring to determine their optimal immunogenicity.

### International guidelines of vaccine development and control: overview

In 2014, WHO developed Guidelines for the evaluation and lot release of typhoid glycoconjugate vaccines, and these were replaced in 2020 by more comprehensive WHO recommendations (WHO 2014a, b, 2020b). Physicochemical tests can ensure batch consistency of conjugate vaccines and were exemplified by the WHO and Pharmacopoeia guidelines based on predecessor conjugate and unconjugated polysaccharide vaccines. Molecular size distribution, the saccharide and protein quantity and their ratios must be considered when determining the structure of the conjugate with appropriate analytical methods. The key testing areas required for vaccine quality control are described below, including the techniques used to measure the relevant structures (Table 7).

**Table 7:** Analytical methods for glycoconjugate characterisation: review table aligned against existing release control guidelines (WHO 2014a, b, 2020a, b)

Control analysed	Routine analytical techniques recommended	Technique can be used to characterise:				Benefits of the technology	Drawbacks
		Monoval. Vi PS	*Shigella* LPS O-antigens	GMMA	Synthetic conj.		
Identity	**Immunological tests:** Immunoblot analysis	Yes	Yes	Yes	Yes	Direct	Need for specific markers for antigens
	ELISA	Yes	Yes	Yes	Yes	Proven to work well with established PS-protein targets	Need for specific markers for antigens In-house ELISA protocols, such as biotinylated Vi ELISA, are generally unable to substitute commercial VaccZyme reads (WHO Expert Committee on Biological Standardization 2018)
	Physicochemical tests: NMR	Yes	Yes	Yes	Yes	Sensitive to small differences such as sugar linkages No calibration is required typically	Instrument intensive Sensitive to small differences such as sugar linkages and the presence of substituents Not easy for the final product (material limitation)
Saccharide content	**Colorimetric methods:** Hestrin assay (hydroxylamine)	Yes	Yes	Yes	Yes	Easy to perform and analyse, low cost Provides information on most common PS components	Fails to differentiate substances with identical composition and variations in linkages Requires immunological methods to be certain of sample identity. Two-step process with spectrophotometric quantitation Not a direct method for PS content, rely on known % of *O*-acetylation
	Resorcinol sulphuric acids Seliwanoff assay (sialic acid)	Yes	Yes	Yes	Yes	Easy to perform Rapid No need to calibrate like with HPLC or NMR Phenol sulphuric acid works with most sugars Scaled down to microplates	Large sample needed Complex sample preparations derivatisations Highly toxic chemicals Low throughput Broad reactivity substantial interference Need to be coupled with quantitative instruments Reagent instability, poor reactivity and undesirable handling of highly toxic reagents (Noyes et al. 2013)
	Phenol sulphuric acid (hexoses/pentoses) (Dubois et al. 1951)	Yes	Yes	Yes	Yes		
	Carbazole assay (uronic acids)	Yes	Yes	Yes	Yes	Can be applied for Vi PS but is not ideal	Polyhexosaminuronic acid structure of VI is resistant to acid hydrolysis and the aminouronic acid moieties do not form the chromophore in the carbazole assay
	Dische colorimetric assay (methylpentoses) (Dische and Shettles 1948)	Yes	Yes	Yes	Yes	Rapid Suits most sugars	Unstable reaction for hexoses with colour change during the reaction Spectrophotometer needed to quantify the amounts of sugars present Uses H_2_SO_4_ that is a harsh method and may decompose some structures Not adequately sensitive
	Acridine orange	Yes	Yes	Yes	Yes	Rapid	Limited to characterisation by the presence of carboxylic groups at a certain spatial distance Accepted for polysaccharide concentration estimation
	Anthrone sulphuric-acid method (Leyva et al. 2008) Anthrone colorimetry for hexoses/pentoses	Yes	Yes	Yes *Not good for multivalent	Yes	Rapid Works best with free sugars	Non-stoichiometric: choice of standard is extremely important for accuracy Harsh, large sample needed Reaction varies with carbohydrate type, not suitable for a mixture of sugars
	**Chromatographic methods:** HPAEC-(PAD/MS/CD)	Yes, with suitable conditions for every vaccine type				WHO TRS Guidelines state superiority of reproducibility over colorimetric methods (acridine orange) Superior method for monosaccharide characterisation in simple formulations	Vi PS is resistant to acid hydrolysis, thus traditional HPAEC-PAD needs modification of the method More difficult to analyse results for multivalent and cross-linked formulations
	HPLC-(UV/SEC)	Yes, but may need method improvement		Yes	Yes	NP-HPLC can be used for *Shigella* monosaccharide analysis (Ravenscroft et al. 2016)	Normal phase-HPLC is limited to analysis of glycolipids and hydrophobic carbohydrate derivatives due to the hydrophobic mobile phase
	**Immunological methods:** Rate nephelometry	Yes	Yes	Yes	Yes	Can be used for multivalent candidates	Not suitable for the lowest amount of PS Need suitable mono- or poly-clone Ab and PS working standard
	Rocket immune electrophoresis	Yes	Yes	Not ideal	Not ideal	Best with monovalent types	Not suitable for all bulk Vi conjugates (WHO Expert Committee on Biological Standardization 2018) Must be optimised to quantitate Vi PS in bulk conjugates, to correct for molecular weight discrepancy between standard and sample
	**Physicochemical tests:** NMR	Yes	Yes	Yes	Yes	Convenient No standards required	Only for the concentration of fully *O*-acetylated polysaccharide Not easy to characterise spectra for conjugates
	GC/MS	Yes	Yes	Yes	Yes	GC/MS provides information on monosaccharide composition and linkages	Prior PS derivatisation required for GC/MS, as polysaccharides are not volatile
Position of *O*-acetylation and *O*-acetyl content	Hestrin assay	Yes	Yes	Yes	Yes	Easy to perform Validated for Vi PS	Not very precise often showing lower *O-*acetyl content (Lemercinier et al. 2000)
	HPAEC-CD (conductivity detection)	Yes	Yes	Yes	Yes	More precise and sensitive than colorimetry (10–20-fold)	No information on the position of *O*-acetylation (Kao and Tsai 2004)
	LS/HR/HR-MAS-) NMR	Yes	Yes	Yes	Yes	Gives structural and position information	Less sensitive with Vi polysaccharides due to the complex structure interactions Full de-*O*- acetylation is required because the NMR peaks attributable to *N*-acetyl and *O*-acetyl in native Vi have the same chemical shift, and so if de-*O*-acetylation is incomplete then the integral of the *N*- acetyl peak cannot be accurately measured May not detect the *N*-acetyl resonance for unconjugated PS (Jones et al. 2020)
	Enzyme immunoassay	Yes	Yes	Yes	Yes	Can be used instead of Hestrin assay as an easy and more sensitive method	Not as sensitive below 50% *O*-acetylation Needs PS-specific antibodies No structural information
	HPLC	Yes	Yes	Yes	Yes	Common method for compositional analysis Can be coupled with RI and NMR	No structural information Depends on the hydrolysis susceptibility of PS
Free PS content: Separation of free PS	Immuno- or detergent – precipitation (ammonium sulphate/ethanol/CTAB/deoxycholate conjugate precipitation)	Yes	Yes	Yes	Yes	Specific to the substrate (in case of immunoprecipitation)	Detergent may deteriorate PS structure if too harsh
	Gel filtration	Yes	Yes	Yes	Yes	Qualitative	Indirect
	Ultrafiltration	Yes	Yes	Yes	Yes	Effective to filter out majority of free PS Quick	Sensitive to pH and buffer type and ultrafiltration behaviour varies with concentration polarisation effects of PS (Emami et al. 2018)
	Isopycnic density gradient ultracentrifugation	Yes	Yes	Potential	Yes	Traditional method for purification of mucin glycoconjugates (see Harding 1992)	Caesium salts need removing by dialysis afterwards. As far as the authors are aware, not yet applied to glycoconjugate vaccines
	Capillary electrophoresis	Yes	Yes	Yes	Yes	Affordable Acceptable precision and linearity Can be used for mixed saccharides	Need for appropriate migration standards and good temperature control
	Reverse-phase Solid Phase Extraction	Yes	Yes	Yes	Yes	Affordable and easy to perform Quantitative	Must consider retention properties for each saccharide (tricky for mixtures) Colorimetric quantification may cause lower precision
Molecular size or mass distribution/molecular integrity	HPSEC-(UV/RI/Fluorescence/RN/RI/DLS/MALS-VISc) gel filtration using: refractive index detector/colorimetric assay or light scattering detector	Yes	Yes	Yes	Yes	Works well for depolymerised Vi saccharide	Loss of molecular integrity is not always seen with hydrodynamic measurements, as the unfolding of a detached PS results in aggregation increasing the hydrodynamic radius of the PS (Ravenscroft et al. 2015b) Cationic interactions with the stationary phase or inappropriate pore size in the column may lead to low PS recovery and bias of the Mw estimation (Bayart et al. 2019)
	SE-/SV-AUC SV-AUC with UV/vis system	Yes	Yes	Yes	Yes	Recognised as a gold standard for assessing molecular integrity and aggregation for mAbs, highly resolving Matrix-free and works with non-inert molecules Provides information on molar mass, homogeneity of sample and sedimentation coefficient distribution No need for a separation matrix	SV-AUC: requires interference optics if insufficient chromophore content in the PS Need for non-ideality correction if the higher concentration is necessary for analysis (low concentration usually used that does not need to be corrected for) Relatively expensive and time-consuming
	A4F-(DLS)	Yes	Yes	Yes	Yes	No issues with column-phase interaction and may serve better for native Vi analysis (but gives similar results with depolymerised PS) Thesis needs statistical confirmation and evaluation of PS concentration effect on solubilisation (Bayart et al. 2019)	Low signal at the detector when below 1 mg mL^−1^ Misleading overlapping signal for high Mw PS at low concentration Preparation of the sample is important and can affect the result (Bayart et al. 2019)
	AFM	Yes	Yes	Yes	Yes	Resolution of single molecules at the nanoscale Versatile and images almost any type of surface Used to measure any force interactions Simple sample preparation	Sample in contact with liquid but not a true solution because it is fixed to a surface
	DLS	Yes	Yes	Yes	Yes	Matrix-free Possible to couple with SEC-MALS Quick Detects aggregation	Very sensitive to larger supramolecular particles (particularly at low angle) as they scatter light disproportionally much more Although current software is good at filtering much of this out, the sample must still be clarified
	SEC-MALS	Yes	Yes	Yes	Yes	Often selected to determine the weight-average molar mass (M_w_) of PS Directly detects the most abundant molecular size population in a wide range of molecular sizes (~ 20 Da–25 MDa) Allows separation of impurities Possible to modify with LALLS at a single low angle (< 10°) to remove the need for extrapolation calculations and couple with RI to get concentration for Mw	For radius of gyration, Mw > 150,000 g/mol is preferable For very large molecules, correct formalism must be applied to get the correct result. Larger molecules (> 1 GDa in size) increase the uncertainty of the measurement as linearity decreases with growing size
	NTA	No	No	Yes	Yes	Detects small molecules Allows size evaluation and number of particles counts Suitable for nanoparticle-based vaccines	A range of parameters need to be adjusted both for video capture (camera gain and shutter speed) and data elaborations (filter settings, background subtraction, removal of blurring, minimum track length, minimum expected particle size and detection threshold) This makes standardisation of the NTA technique difficult and is strongly operator dependent Does not determine conjugation sites (Giuntini et al. 2017)

#### Identity and compositional analysis of polysaccharide-based vaccines

The identity of a glycoconjugate vaccine for enteric pathogens can be defined in monosaccharide composition, *O*-acetyl group quantitation and purity of the sample. ^1^H-nuclear magnetic resonance is validated as a sole routine release method for PS identity and purity, which is recommended by the United States Pharmacopeia (USP) and WHO for *S.* Typhi Vi PS and is a superior technology to less precise colorimetric techniques (although it cannot measure residual nucleic acid or protein) (Lemercinier et al. 2000; WHO 2017b; United States Pharmacopeial Convention 2018). NMR provides data on the PS structure, stability and degradation pathways, and can be used with a quantitative standard as a qNMR method to get relative molecular mass and content (WHO 2017a). Uni-dimensional and two-dimensional NMR spectroscopy can be used for *S.* Typhi and *Shigella* O-Ag in either glycoconjugate or GMMA preparations, and is a relatively rapid and information-rich characterisation method (Berti and Ravenscroft 2015; Raso et al. 2020). NMR spectroscopy has also proven valuable for the detection, identification and quantification of process impurities in the polysaccharides (Xu et al. 2005; Beri et al. 2019). Nevertheless, spectra are less resolved for the hydrophobic and rigid structure of tightly packed substituents in the sugar ring of Vi due to the rapid spin-spin relaxation rates, which is not ideal for differentially *O*-acetylated serotypes (Lemercinier et al. 2000).

Other methods for Vi polysaccharide quantitation include an acridine orange dye colorimetric method (Stone and Szu 1988), rate nephelometry and rocket immunoelectrophoresis (WHO 2017b). High-performance anion-exchange chromatography-pulsed amperometric detection (HPAEC-PAD) is a common technique applied to quantitate Vi saccharide content in drug substance and drug product using an alkaline hydrolysis method (Micoli et al. 2011). Measuring the Vi content of conjugated or non-conjugated polysaccharide vaccines by six laboratories with HPAEC-PAD resulted in good inter-laboratory comparability and produced improved results distribution of Vi content when the homologous Vi PS standard was used to quantitate the Vi content in a vaccine from the same source (*Citrobacter freundii* or *S.* Typhi). The rocket immuno-electrophoresis was performed by four laboratories but could have limitations when used for bulk conjugates, due to the standard-sample molecular mass disagreement. It, however, gave good results for the PS alone (WHO 2017b; Gao et al. 2019).

LPS and glycan composition in *Shigella* can be determined with the methods described above as well. The ^1^H diffusion-ordered spectroscopy (DOSY) NMR coupled with HPLC-SEC (size exclusion chromatography) with differential refractive index (dRI) can be used to detect LPS chain length, size and structure variations in *S. flexneri* GMMAs. The number of LPS core reducing end KDOs (2-keto-3-deoxy-octonate) is isolated and quantified by HPLC-SEC as well. The percentage of O-Ag can be determined as the molar ratio of their KDO to total KDO. O-Ag sugar content is also possible to quantify using the Dische colorimetric method (methyl pentoses (6-deoxyhexoses)) (Dische and Shettles 1948). Colorimetry is less precise so physical or immuno-detection methods may be prioritised. Total *O*-acetyl content, as well as *O*-acetyl content variations, can be confirmed with 2D NMR (Raso et al. 2020). NMR and HPLC-SEC are often applied to check glycoconjugate purity as well (Berti and Ravenscroft 2015; Ravenscroft et al. 2015b). HPAEC-PAD run with appropriate standard or standard mixtures can be applied for GMMA or complex O-Ag saccharide chains to calculate the relative molar concentration of glucans (Gerke et al. 2015).

The number of polysaccharide chains bound to the carrier protein can be deduced with limited information if chain lengths are known; however, these methods do not give information on their position. The conjugation positions can be identified by mass spectrometry, but it is still a challenge to map conjugation sites in populations of polydisperse molecules. Some reports on capsular polysaccharides (CPS) show that solid-state NMR spectroscopy is a working alternative to determine PS/protein conjugation pattern and the degree of conjugation; however, it has not yet been reported for *Salmonella* Typhi, iNTS or *Shigella* conjugated vaccines (Giuntini et al. 2017). To determine sugar:protein ratio, colorimetric tests are still popular due to their low cost and relative simplicity, for example anthrone and a colorimetric protein concentration assay (Leyva et al. 2008; Ravenscroft et al. 2015b).

Standardised ELISA methods are available for immunogenicity determination. Currently, two methods have been accredited as repeatable and reliable for serological ELISAs of serum responses against Vi-based conjugates from clinical studies: commercial VaccZyme and in-house Vi-poly-l-lysine (PLL)-based immunoassays, where Vi is pre-coated with PLL to give superior results to native coating, as PS is negatively charged allowing PLL to adhere and improve Vi interaction with solid phase (Rijpkema et al. 2018; Rigsby et al. 2020). The biotinylated Vi ELISA can be used for control comparison of potencies but not for the geometric mean potencies, or potency estimates, as it gives alternative reads to the VaccZyme ELISA (Rijpkema et al. 2018). Following the request of the WHO Expert Committee on Biological Standardization (ECBS), Vi-PLL ELISA has been finally assessed in 2020 as a suitable substitute to the VaccZyme ELISA, allowing to use this uniform non-commercial, and thus more available, method to use at a lower cost. The Vi-PLL procedure is publicly available and uses standard biological agents, as opposed to VaccZyme (WHO 2018; Rigsby et al. 2020). Key considerations for implementation and smooth performance are the level of reagent standardisation (reference standard), validity criteria (system and sample suitability) and the inclusion of assay-specific run controls which may be study samples representative of Vi PS vaccines (Rigsby et al. 2020).

#### Determining *O*-acetyl content

The most basic, cheap and straightforward method for *O*-acetyl content estimation is the colorimetric Hestrin assay (Hestrin 1949) which is routinely used with purified glycoconjugate vaccines or polysaccharide components but gives less precise information (Gao et al. 2019). Another potential approach to determine the degree of *O*-acetylation could be an enzyme immunoassay using an *O*-acetyl-specific monoclonal antibody. However, due to the importance of the *O*-acetyl pattern for vaccine immunogenicity, complementary methods to colorimetry and immunoassays were developed, enabling acetyl group detection, content and positioning on the purified CPSs. These are currently NMR, and HPAEC with conductivity detection (HPAEC-CD), although this is not yet widely used (Lemercinier et al. 2000; Kao and Tsai 2004; Hitri et al. 2019). NMR is considered to give more information on *O*-acetyl position in general and has benefits over wet chemical methods since it can be used for CPS fingerprinting and gives a wider spectrum of information; however, it becomes difficult to interpret when more complex cross-linked conjugates are evaluated. Nevertheless, ^1^H and ^13^C NMR coupled with mass spectrometry was applied successfully to detect *O*- and *N*-linkages in Vi PS in a GMMA construct (2020). HPAEC-CD is up to 20 times more sensitive compared with the Hestrin test, requires a smaller amount of material compared to NMR and is a superior method to evaluate monosaccharide composition (Mulard 2017).

While circular dichroism (CD) is commonly applied for the characterisation of carrier protein integrity, effect of conjugation and stability determination, a recent study has shown its potential for monitoring *O*-acetylation (Jones et al. 2020). A strong signal from the Vi, unusual to polysaccharides, is observed in the far-UV from *O*-acetylated and non-*O*-acetylated residues in the Vi. With a larger amount of information generated by CD for Vi compared to other conjugates, it may become a useful method to monitor vaccine stability by analysing the degradation or de-*O*-acetylation behaviour of the polysaccharide. This method poses larger uncertainty when reading weak spectra. To assess the suitability of CD for this application, more detailed data comparisons from orthogonal methods and corrections for specific saccharides are required (Jones et al. 2020).

#### Detection of polysaccharide degradation or detachment in vaccine products

Free, unconjugated PS content is the main indicator of vaccine stability and inversely correlates with its potency, or effective dose; a product with increased free saccharide content could be less immunogenic, due to a lower amount of conjugated saccharide, and the potential for unconjugated saccharide to neutralise pre-existing antibody. It can be quantified successfully by only a limited number of methods that allow reproducibility. This measurement is reflecting the stability of the glycoconjugates. To determine free PS content, two steps are required: free PS separation and its quantification. The first step can routinely be done by chromatography (size, ion-exchange, hydrophobic interaction), chemical precipitation with acid or detergents, ammonium sulphate or ammonium adsorption, capillary electrophoresis, gel filtration, centrifugal ultrafiltration, ultracentrifugation, solid-phase extraction or immunoprecipitation. The second step can be performed using colorimetry or immunoblotting or by using physicochemical techniques (HPLC, HPAEC-PAD or gas chromatography (GC) post-hydrolysis), which are more robust approaches (Ravenscroft et al. 2015b).

**Fig. 1:**
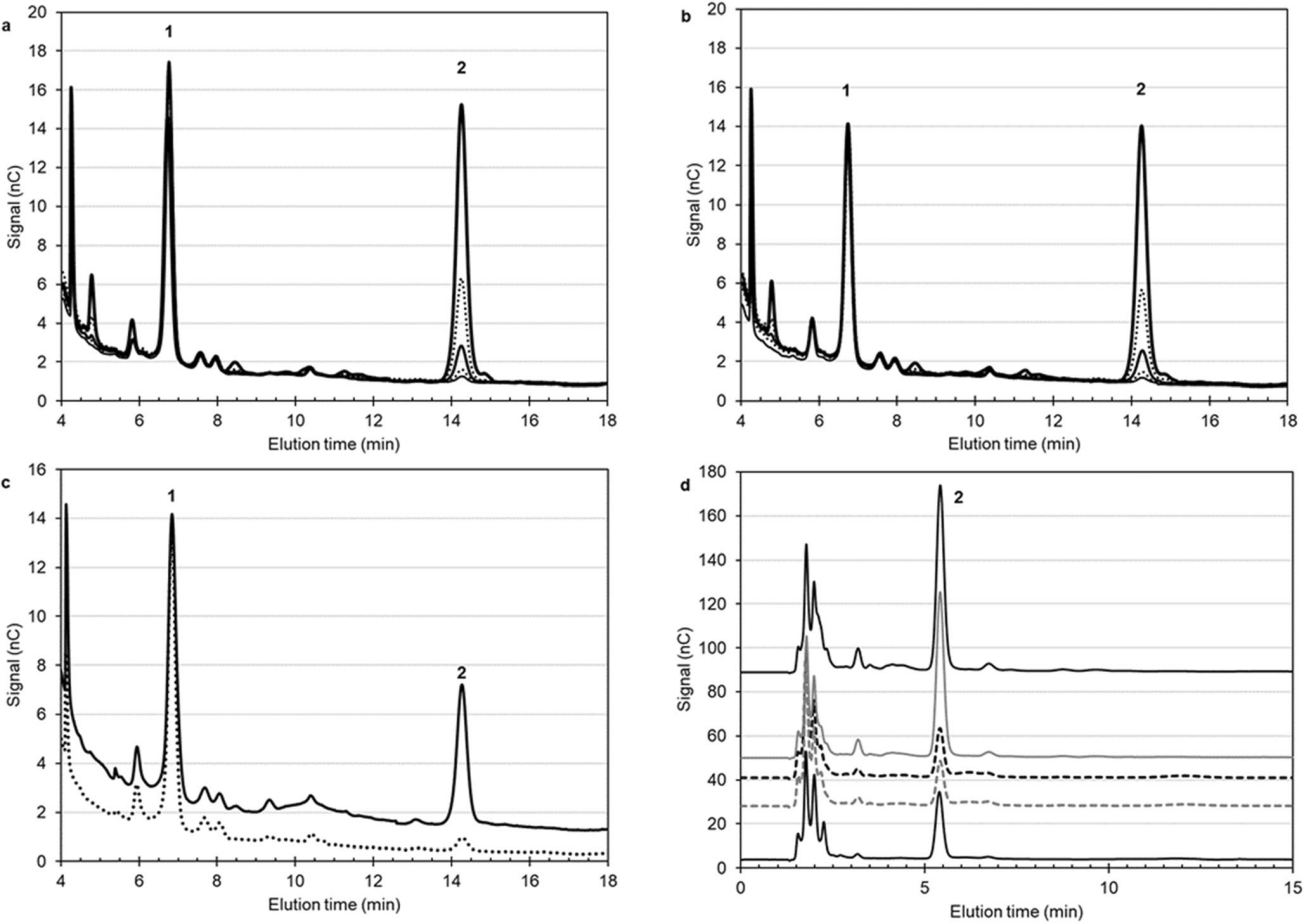
HPAEC-PAD chromatograms obtained from NaOH-depolymerized (**a**–**c**) and TFA-HCl-hydrolysed (**d**) *C. freundii* Vi polysaccharide (**a**), *S*. Typhi Vi (**b**) polysaccharide and a typhoid conjugate vaccine (**c**) The individual traces are described in the text

Giannelli et al. found that UV-coupled HPLC-SEC detection of free Vi PS proved to be problematic due to the variable extinction coefficient of Vi that depends on *O*-acetyl content and chemical modifications required for carrier attachment (2017). An alternative free PS purification method was introduced using the Capto Adhere resin separation that worked irrespective of conjugated protein and delivered a more sensitive and quantitative measurement for a range of Vi lengths. It works by entrapment of intact conjugates and HPAEC-PAD quantification of the Vi component recovered in the filtrate. Capto Adhere resin separation coupled with HPAEC-PAD achieved 75–120% recovery of free Vi PS. The absence of conjugates in the buffer strip solutions was verified by a dot-blot assay and HPLC-SEC chromatograms (Giannelli et al. 2017). Their most recent paper on Vi detection suggests an optimised hydrolysis conditions for the HPAEC-PAD detection method to get more accurate data on the de-*O*- and de-*N*-acetylated Vi PS monomer, discussed later in the review. A deoxycholate (DOC) precipitation method (Lei et al. 2000), in which free saccharide is recovered in the supernatant of DOC- precipitated protein samples, has also been shown to measure reproducible % free saccharide levels (Fig. 1).

Another one-step immunoprecipitation method based on anti-carrier protein antibody-coupled Sepharose beads was developed at the Serum Institute of India. The method claims to overcome HP-SEC limitations and can be applied for free PS from multivalent polysaccharide-protein conjugate vaccine formulations, total PS content and percent adsorption of polysaccharides (Serum Institute of India 2013).

#### Molecular size profiling and solution properties of glycoconjugate vaccines

Glycoconjugates manufactured by conjugating PS to a carrier protein will have increased mass, altered shape and possibly changes in behaviour in solution compared to their individual components. Flexible interactions in solution and inter-molecular interactions can be influenced by the character of the polysaccharide as well as the protein, and changes to protein sites required for antigen processing and T-cell epitope accessibilities could be affected. The type of carrier protein that is conjugated may influence the choice of analytical techniques. While monovalent “sun type”, for example CRM_197_ conjugates may be more straightforward to characterise, TT-based cross-conjugated glyco-vaccines (e.g. “lattice type”) may be more challenging (Barel and Mulard 2019). Proven methods to measure molecular size distribution, hydrodynamic size, viscosity and solution behaviour (of the glycoconjugate or the separate polysaccharides and/or carrier proteins) include SEC-MALS/UV/RI/Viscosity, degree of polymerisation (repeating unit analysis), analytical ultracentrifugation and optical spectroscopy (MacCalman et al. 2019).

When determining molecular integrity of polysaccharides in solution, challenges linked to the polydisperse nature of the saccharides arise, in addition to their higher non-ideality (through co-exclusion and polyelectrolyte effects) compared to proteins. SEC-MALS is considered a useful technique with minimal non-ideality effect due to the dilution from the columns. However, it relies on interaction with a matrix, is dependent on % recovery and sometimes ignores hydrodynamic changes brought about by the PS moiety detachment or protein unfolding which increases the apparent relative size, as if the conjugate structures were intact (Ravenscroft et al. 2015b). In addition, the separation columns have a size separation limit, and the assumption must be made; there are no anomalous interactions with the polymer matrix which makes up the separation columns. SEC-MALS/RI/viscosity of Vi polysaccharides in different eluents demonstrated the need to consider column choice and buffers salts to obtain representative recoveries and highlighted the differences that salt composition makes to hydrodynamic size and viscosity (Hitri et al. 2019). See also the following examples of other vaccine-relevant oligo- and poly-saccharide components (Jumel et al. 2002; Bardotti et al. 2008; Harding et al. 2012; Lockyer et al. 2015).

Analytical ultracentrifugation (AUC)—a matrix-free method—has also been extensively applied for molecular distribution and molecular weight of polysaccharides (see, for example Harding et al. 2010 and 2015 & references cited therein). The two main types of AUC are sedimentation velocity and sedimentation equilibrium. Sedimentation velocity provides separation based on size and shape, and analysis, normally from Rayleigh interference optical records. Sedimentation coefficient distributions provide a measure of heterogeneity, and if conformational information is known, the distributions can be converted into molecular weight distributions using the “Extended Fujita” method. Sedimentation equilibrium—at slower speeds than for sedimentation velocity—gives optical records that are directly related to molecular weight. Some caution is necessary with regard to non-ideality, either by working at low concentration to minimise these effects (the lowest working concentrations are ~ 0.05 mg/mL for sedimentation velocity and ~ 0.4 mg/mL for sedimentation equilibrium) or by making measurements at different concentrations and performing an appropriate extrapolation to zero concentration where non-ideality effects vanish. Conformation and flexibility can be estimated using power-law coefficients (relation between sedimentation coefficient and molecular weight) and the Wales-van Holde ratio (ratio of the “non-ideality” concentration dependence sedimentation coefficient parameter to the intrinsic viscosity) and using the persistence length calculations based on “HYDFIT” combinations of SEC-MALS, viscosity and AUC data (see Abdelhameed et al. 2012, 2016a, b) for examples with Hib and meningococcal-TT conjugates.

Optimal spectroscopic methods such as *near*-UV CD and Trp fluorescence spectroscopy also can report on the mobility and solvent accessibility of aromatic amino acid side chains. The effects of conjugation on these groups, as well as the secondary structure and folding of a carrier protein, as determined by *far*-UV CD, are important characterisation methods and will remain important for the initial characterisation of novel recombinant carrier proteins. Carrier protein and conjugate molecule characterisation and assessment of thermal stability have been described for these carrier proteins: CRM_197_ (Crane and Bolgiano 1997; Bolgiano et al. 2001; Ho et al. 2001; Bardotti et al. 2008), TT (Ho et al. 2002; Lockyer et al. 2015), rEPA (Ho et al. 2006), DT and protein D (Lockyer et al. 2015). Structural effects on conjugated carrier proteins have been demonstrated to correlate with reduction in the immune response against the carrier (Beresford et al. 2017).

### Novel *Shigella* vaccines: glycoconjugates and GMMA

Several vaccines in clinical trials are currently targeting the most common serotypes of *Shigella* (Table 5); however, a more promising direction to take maybe towards multiprotection formulations (WHO 2020a). The WHO suggested existing recommendations for glycoconjugate vaccines control to be followed in product guidance for *Shigella* vaccines (WHO 2020a). Since *Shigella* vaccines are based on the O-polysaccharides anchored to the LPS, they are more challenging to produce and control. Conjugation chemistry of multiple antigens is structurally complex, and LPS requires laborious extraction and detoxification, which may affect molecular integrity. LPS and lipoproteins in GMMA act as immunostimulatory components, which alter their reactogenicity: the level of LPS acylation is important for determining the potency and safety of vaccines (De Benedetto et al. 2017a).

Alternatives to multistep chemical conjugation include bioconjugation using transformed *E. coli*, GMMA vesicles and synthetic conjugates (Kis et al. 2018). GMMA vaccines are already manufactured in GMP-quality conditions against *S. sonnei*, *S. flexneri* and nontyphoidal *Salmonella* strains (Gerke et al. 2015). Novel GMMA formulations are usually characterised by conventional analytical methods used for other glycoconjugates, being verified for their use (Micoli et al. 2020a, b; Raso et al. 2020). For GMMA, O-Ag core sugar content can be quantified by HPAEC-PAD, after performing acid hydrolysis directly on GMMA. After O-Ag extraction, total sugar content can be done using a suitable colorimetric method, or NMR, which also provides structural information and assesses purity (Raso et al. 2020). The concentration of O-Ag can be determined with a combination of NMR and HPAEC-PAD (Gerke et al. 2015). Protein and lipid content will need to be determined to calculate LPS/protein ratios.

The GMMA size distribution can be determined by dynamic light scattering (DLS). Other methods described for GMMA particle size include multiangle light scattering (MALS) that can be coupled to the high-performance liquid chromatography–size exclusion chromatography (HPLC–SEC/MALS, which also helps to check sample purity) and nanoparticle tracking analysis (NTA) (De Benedetto et al. 2017b).

Meloni et al. used hydrolysed GMMA (1% acetic acid v/v, neutralised with 28% NH_4_OH) from *Salmonella* Typhimurium SL1344 Δ*tolR* O-antigen (O-Ag) PS purified by a tangential flow filtration method to separate and analyse O-Ag, and it was revealed that such method strips off the low molecular mass O-Ag completely in GMMA and wild-type bacteria, shown by HPLC analysis of the batches (2015). Saccharide content was then analysed by HPAEC-PAD, confirming the differential monosaccharide ratios of low and medium molecular mass antigens in GMMA and wild-type bacteria. This O-Ag has more similarity to an LPS, which may be relevant for *Shigella* GMMA characterisation. This method highlights that O-Ag purification must be optimal to fully characterise GMMA constituents; however, the approach may be useful to evaluate the proportion of larger Mw components with HPAEC-PAD, and seems to work well as a direct O-Ag separation method in enteric *Salmonella* (Micoli et al. 2013).

Ravenscroft et al. described analytical techniques used to prove the structural integrity of *S. dysenteriae* type 1-EPA glycoconjugate produced by innovative biosynthetic *Escherichia coli* glycosylation that are suitable for characterisation of other *Shigella* serotypes and based on conventional approaches like (RP/NP)HPLC-SEC/UV, immunoblot detection, 1D and 2D NMR and MALDI MS/MS post-trifluoroacetic acid (TFA) hydrazinolysis (2016). The recovery of the Rha repeating unit was low as the free 6-deoxy hexose decomposed due to hydrolysis employed to release GlcNAc, suggesting the importance of optimal hydrolysis conditions. The *S. dysenteriae* type 1 O-PS structure was fully characterised by 1D and 2D NMR spectroscopy with full information provided on the Gal, two Rha and GlcNacspin systems evaluated by the use of 1D TOCSY, 2D ^1^H–^1^H (COSY and TOCSY) and ^1^H–^13^C (HSQC, HMBC and HSQC-TOCSY) experiments (Ravenscroft et al. 2016).

## Part III

### Emerging techniques to aid glycoconjugate design

#### Structural vaccinology

Structurally complex polysaccharides conjugated to carrier proteins may pose a risk of exerting immunodominance over a less immunogenic but essential epitopes in a combination vaccine, which is highly undesirable for immunisation. Neoepitopes that form due to degradation or modification of polysaccharides may adversely impact the immunogenicity of native polysaccharides. Getting access to comprehensive structural evaluation of the mechanisms of binding and epitope characterisation tools may help to expand current understanding of how immunodominance is dictated in glycoconjugates and even provide novel data allowing for the enhancement of the antigen structure to achieve adequate and balanced protection (Anish et al. 2014).

#### Computational methods to facilitate synthetic antigen design

Establishing and evaluating carbohydrate structure-function relationships that affect immunogenicity are topics of high interest. Glycan shape complexity, chain flexibility, dynamic properties of pyranose rings and diversity of functional groups make structural analysis a priority that would benefit from multiple approaches. Where 3D physicochemical methods are not informative enough, computational methods may supplement the characterisation of structural determinants of affinity, specificity and antigenicity. In silico simulations could allow for a study of conjugation and binding at an atomic level when they cannot be experimentally deduced. Computational methods allow for tracking molecular effects on saccharide antigenicity (through enthalpy and entropy changes), which is important for the selection of the right candidate for synthesis. Moreover, currently used methods, such as X-ray diffraction or NMR spectroscopy, may provide biased results for large saccharides in more dilute solutions. While NMR techniques are relevant for homogeneous saccharide characterisation, implications linked to molecular mass limit the scope of applications for large antibody-PS complexes and often need coupling with in silico molecular dynamics (MD) simulations for structural definition (Anish et al. 2014). MD simulations of vaccine-relevant PS that consider chain length and counterions in a hydrated environment have identified potential epitopes and conformer distributions for Vi PS and O-Ag (Hitri et al. 2019; Hlozek et al. 2020).

Characterisation and structural information for glycoconjugates cannot be achieved by a single method; thus, multiple biophysical techniques combined with computational methods allow deduction of carbohydrate structure-function analysis and antibody interaction (Anish et al. 2014). Molecular modelling coupled with NMR spectra of the molecular structure has been implemented for more optimal design and immunogenicity of *Shigella* SD1 synthetic glycoconjugates. It allowed identification of a preferred hairpin conformation of a synthetic O-Ag (the ABCD tetrasaccharide) that was representing the native O-Ag more closely. A hairpin conformation for ABCDA’ saccharide and larger O-Ag parts in a helix conformation allowed partial exposure of B and D monosaccharides, thereby inducing antibody recognition that could lead to an enhanced anti-O-Ag antibody response, which is a key parameter for vaccine potency and safety considerations (Barel and Mulard 2019). These methods provide alternatives to the 3D structural analysis of polysaccharides by small-angle X-ray/neutron scattering, X-ray crystallography and NMR (Anish et al. 2014).

### Current efforts for better polysaccharide characterisation

#### Modified methodologies of HPAEC-PAD for more precise Vi saccharide quantitation

HPAEC-PAD is currently the most robust and detailed quantification technique used for PS compositional analysis of bacterial vaccines (Rohrer 2020). However, due to the low susceptibility of Vi and other uronic acids to PS depolymerisation, this method is constantly being optimised for more accurate and precise readings. While strong alkaline-based depolymerisation is efficient for Vi in monovalent formulations, combinations with *S. sonnei* O-antigen, where co-elution with Vi may occur, are likely coming from the degradation of the alturonic acid (Gerke et al. 2015; Giannelli et al. 2020). Giannelli et al. further improved the hydrolysis step for HPAEC-PAD by suggesting acid hydrolysis with simultaneous use of trifluoroacetic and hydrochloric acid (TFA:HCl in a 2:13 v/v ratio), to prevent such co-elution and verified product formation using ^1^H NMR, ^13^C NMR, COSY and HSQC (Giannelli et al. 2020). The presence of 2-amino-galacturonic acid monosaccharide in equilibrium between α and β conformations was also observed for Vi. Recovery of 101% was reported of reduced (de-*O-* and de-*N* acetylated) monosaccharides with increased sensitivity due to the high yield of hydrolysis product(s) compared to the depolymerisation method. This procedure could facilitate the development of standards in enteric vaccines containing both Vi PS and O-Antigens that also contain amino uronic acids (Giannelli et al. 2020).

We have compared the novel HCl:TFA hydrolysis method for Vi PS quantitation of a typhoid conjugate vaccine containing *S.* Typhi Vi PS, with the standard NaOH hydrolysis conditions (Gao et al. 2019) using WHO International Standards for the polysaccharides (Fig. 1).

The content of Vi saccharide in a typhoid conjugate vaccine was quantified using WHO International Standards for Vi PS (Gao et al. 2019). Standards and vaccine samples were base depolymerized with 2 M sodium hydroxide for 4 h at 110 °C (panels a to c) or acid-hydrolysed with 10% TFA-8 M HCl for 4.5 h at 80 °C (panel d), following the recovery of unconjugated PS in the supernatant of a vaccine sample precipitated by sodium deoxycholate (1% w/v) pH 8.0 and HCl (0.04 M) (Lei et al. 2000). The separation was performed on a CarboPac PA-1 column (2 mm) at 25 °C with an Amino Trap as a guard column. Base-treated samples were eluted at a flow rate of 0.25 mL/min for 30 min, and with elution conditions of 0–2 min, 100 mM NaOH, 40 mM NaNO_3_; 2–22, 100 mM NaOH, 40–150 mM NaNO_3_ (Gao et al. 2019); acid-hydrolysed Vi was run at 0.375 mL/min using isocratic elution with 400 mM NaOH for 15 min. *N*-Acetyl glucosamine-6-phosphate (10 μg/mL) (peak 1) was injected in samples and standards to normalise the Vi signal in base-treated samples. Standard curves were constructed using 27, 9, 3, 1 and 0.5 μg/mL Vi PS shown in panels a and b as alternating solid and dotted lines (peak 2) from *C. freundi*i (panel a) or *S*. Typhi (panel b) polysaccharide samples. The vaccine sample (panel c) injected was 10 μg/mL Vi PS to quantify free, unconjugated (dotted line) or total Vi PS (solid line). Panel D of acid-hydrolysed standards and sample shows from top to bottom, 27 (μg/mL) *C. freundii* Vi, 27 *S.* Typhi Vi, 9 *C. freundii* Vi, 9 *S.* Typhi Vi and total saccharide vaccine sample. Samples were injected in duplicate. For base-treated samples, Vi eluted within the expected time interval, at about 14.3 min, and both standards yielded *R*^2^ > 0.98. For acid-hydrolysed samples, Vi eluted at about 5.4 min and the signal was 6 times higher.

Both the Vi PS concentration (μg per dose) and % free Vi PS were lower using the *C. freundii* sample by 7.7% and 4.3%, respectively, compared to native Vi PS standard under base treatment. This suggested that *C. freundii* is an imperfect alternative to the native Vi that could underestimate Vi content in the sample. Homologous source Vi PS should be used for quantitation (Gao et al. 2019). Linear standard curves were obtained (up to 10 ug/ml Vi) with the acid hydrolysis method, which has the potential to separate amino-uronic acids and O-LPS with higher sensitivity. The establishment of an acid-stable internal standard for quantitation is required.

#### Methods for robust characterisation of multivalent combinations and *Shigella* antigens

*Shigella* LPS and O-Ag analysis may be a laborious task due to a vast diversity of antigens in different serotypes. Currently, primary testing to identify the O-Ag usually limited to techniques using specific identifiers for the serotype detection, such as membrane insertion and ELISAs (Stromberg et al. 2017). The lack of sensitive and selective antigen ligands suitable for every serogroup remains a major problem. Ravenscroft et al. also applied capillary gel electrophoresis for *Shigella dysenteriae* 1-EPA bioconjugates mentioned above to get better resolution of individual repeating units, as well as to monitor their integrity (Ravenscroft et al. 2016).

#### Combination methods suggested for future analysis and vaccine design

Coupling high-resolution mass spectrometry and ^1^H NMR techniques was tested with *Neisseria meningitidis* serogroup B, enabling simultaneous analysis of conjugation reaction course and final products (Yu et al. 2018). However, the utilisation of mass spectrometry with polydisperse macromolecules (10–100 kDa), like *S.* Typhi O-PS, provides less information as in the case of glycoconjugates derived from synthetic antigens or less complex glycans (Méndez et al. 2018).

The NEWCARBOVAX project was launched in 2017, which showcases an effective vaccine design platform that has been tested with CPSs from *S.* Typhi, Hib and type Ib group B *Streptococcus*, that successfully induced T helper cells (T carbs) successfully (CORDIS | European Commission 2020). This platform is going to be a helpful approach if coupled with research into novel conjugation chemistry approaches in this field.

Any of the new and existing conjugates under development may benefit from the application of Quality by Design principles in the development and testing of vaccines and intermediates. Current technologies discussed in this review are focused on more common glycoconjugate formulations. Nanoparticle-based vaccines, protein capsular matrix vaccines and liposomal encapsulation of polysaccharides are becoming a new trend in vaccine design and will require new validated methods of quality control.

Lastly, classical approaches to evaluating thermal unfolding and stability for lipid-based vaccines destined for hot climates may need to be reconsidered to account for their membrane dynamics. The lipid composition of the membranes may affect membrane phase transitions at relatively lower temperatures than expected for the polysaccharides and some protein domains, and the impact of such dynamics on immunogenicity, safety and protection will need to be considered as shown for *Salmonella* and *Shigella* GMMAs using differential scanning calorimetry in combination with monoclonal antibody binding and immunogenicity (Palmieri et al. 2021).

## Summary

Limited antibiotic treatment options against the emerging burden of typhoid fever and shigellosis infections and the continuing need for cleaner water, hygiene and sanitation emphasise the urgent need for effective preventions against communicable enteric diseases. Glycoconjugate vaccines could become a promising solution targeting the most infectious strains of these bacteria. With the updated WHO recommendations on the quality, safety and efficacy of typhoid conjugate vaccines adopted by the ECBS in 2020 and SAGE recommendations for typhoid conjugate vaccines to be used as preventive measures in endemic countries or where there is a high burden of antimicrobial resistance (WHO 2017a), analytical methods for evaluating typhoid conjugate vaccines are becoming established. The efforts to produce protocols for *Shigella* and non-typhoidal *Salmonella* vaccine development will be supported by academia, institutions, public health bodies and funding organisations over the next few years (NIBSC 2020). These are major steps towards faster commercialisation of glycoconjugates and more complex vaccines to address an emerging global need for enteric disease prevention.

This review highlights applications of conventional methods of glycoconjugate and more complex vaccine structures. Alterations to experimental conditions are constantly made and protocols are being modified together with reference standards to serve the demand for higher accuracy for glycoconjugates that are less susceptible for hydrolysis or behave differently in solution. Together with modern computational approaches, a more precise design of glycoconjugates is possible, which allowing better predictions on vaccine behaviour, structural changes in solution and immunogenicity, which can be applied to a broad spectrum of vaccines. More validation is required to establish the most effective and suitable methods for glycoconjugate and more complex vaccine analysis to bring uniformity to existing protocols, aiding future achievements in this area of research.
